# Special Issue “Hepatitis B Virus Infection: From Diagnostics to Treatments”

**DOI:** 10.3390/v12121366

**Published:** 2020-11-30

**Authors:** Thomas Tu, Mark W. Douglas

**Affiliations:** 1 Storr Liver Centre, Westmead Institute for Medical Research, University of Sydney and Westmead Hospital, Westmead, NSW 2145, Australia; 2 Marie Bashir Institute for Infectious Diseases and Biosecurity, University of Sydney, Westmead, NSW 2145, Australia; 3 Centre for Infectious Diseases and Microbiology, Westmead Hospital, Westmead, NSW 2145, Australia

In this Special Issue, we have brought together a broad range of studies on hepatitis B virus (HBV) covering diagnosis, pathogenesis, monitoring, and treatment. In doing so, we have highlighted the many challenges that remain unaddressed, including limitations in technical, virological, clinical, and public health-related resources.

*HBV diagnosis and living with HBV*. Although highly sensitive, specific, widely available, and inexpensive tests are now available to diagnose HBV infection, Freeland et al. highlight some of the major hurdles that still prevent affected people from entering the health care system [1]. They focus on African immigrants to the United States and find (as with many other communities) that stigma and discrimination play a key role in preventing patient engagement in diagnosis and treatment.

Tu et al. further explore these issues in a review of the non-physical impacts of diagnosis on people living with hepatitis B [2]. They combine academic literature with first-hand accounts of lived experiences to show that chronic HBV infection affects more than physical health, with significant psycho-social impacts on those affected.

One major impact on the mental health of people living with hepatitis B is the fear of disease progression. Fortunately, there is renewed interest in updating HBV treatment guidelines, which could reduce this fear dramatically. Hall et al. review recent evidence for early initiation of antiviral therapy [3], which could empower people who do not meet current guidelines for treatment and thereby decrease their fear of disease progression. They also provide an update on when to stop nucleo(s/t)ide analogue treatment while maintaining virological response, a highly-anticipated goal for those currently on life-long antiviral therapy.

*Pathogenesis.* Most HBV-associated liver disease appears to be linked to aberrant expression of viral proteins (e.g., mutated HBV surface or x proteins, HBsAg or HBx) or to dysregulated, chronically activated immune attack of infected cells. Lee et al. [4] focus on HBx as a potential contributor to HBV-associated liver cancer. Here, they find that over-expression of HBx induces downregulation of selenium binding protein 1, which is reduced in many cancers, including (as they report) HBV-associated HCCs. However, the underlying mechanisms by which HBx effects these changes in the chronically infected liver remain unclear.

With respect to the dysregulated immune response, Vinhaes et al. use high-dimensionality analyses of inflammatory perturbation to compare immune markers in the blood of people with chronic HBV infection and those who have undergone spontaneous HBsAg-seroconversion [5]. They find significant differences not only between chronic HBV and HBsAg-negative states but also between people who have cleared HBV infection and those who are HBV-naïve. While it is yet to be demonstrated if any of these markers are linked to HCC risk in patients who have cleared HBsAg, the observed immune perturbation in people who have undergone spontaneous “functional cure” may inform treatment endpoints.

*Systems for developing HBV treatments.* To limit viral pathogenesis and disease progression, HBV researchers continue to direct substantial efforts into designing novel treatments. To facilitate drug development, Jo et al. describe a simple, high-throughput method for quantifying HBV DNA [6]. Their pipeline is adaptable to 96- and 384-well formats and can quantify both intracellular and extracellular (secreted) viral genomes. Their method is highly reproducible and determined the correct IC50 for several compounds with known anti-HBV effects. This assay will undoubtedly prove useful to screen large libraries of compounds to identify novel antivirals.

When developing new HBV treatments, judicious use of model systems is essential to predict which strategies are likely to work in the clinic. Almost all research into antiviral compound discovery and mode-of-action confirmation relies on in vitro systems. Most of this work uses only the well-established “Galibert” clone of HBV (Genotype D). In their review, Kinge et al. remind us that most HBV infections occur with non-D genotypes and give an overview of systems that can be used to study the different strains of the virus [7]. In the same vein, Velkov et al. present a detailed snapshot of the variability of HBV throughout the world, using public sequence databases [8]. They show that not only HBV genotypes, but also serotypes and clinically-relevant mutations vary in frequency around the world. Together, these papers emphasise the need for studies to confirm the pan-genotype antiviral activity of any new therapeutics, so they can be used in all countries and patient populations.

In vitro systems inevitably have limitations in simulating the complex multi-system interactions that take place in a person who is infected. Wettengel and Burwitz provide a timely review into new in vivo models that are being or have been developed to study HBV infection [9]. An immune-competent, HBV infection in vivo model that recapitulates human disease (including liver cancer and progressive liver fibrosis) is the holy grail of preclinical HBV research. In this review, the authors highlight how recent insights into factors that restrict viral infection and replication (e.g., the HBV receptor, NTCP) have now put this dream within reach.

*Approaches to HBV cure.* This Special Issue includes several reports identifying key points in the HBV lifecycle that could be targeted by future therapies. Duraisamy et al. outline current and upcoming treatments in their broad review [10]. They also give a forward-looking perspective on the possibilities of personalised medicine in the management of chronic hepatitis B.

A functional cure (loss of circulating HBsAg) is seen as the most achievable goal for future treatments. One strategy to achieve this is to directly silence viral transcription. Turton et al. review the interactions between cellular transcription factors and viral gene expression [11], highlighting promising therapeutic targets to induce a functional cure.

Other reports identify alternative virus-host interactions that could be targeted. Hossain et al. review the reported interactions between HBV proteins and mitochondrial signalling, with particular focus on innate immunity and viral replication [12]. They highlight several reported interactions between HBV and mitochondria that could be targeted to inhibit HBV replication, although exactly how this can be achieved remains unclear and is the subject of ongoing research.

HBV replication depends on the viral polymerase, so it is an obvious target for antiviral therapy. Drugs targeting its reverse transcriptase function were developed not long after the discovery of HBV, but recent research has looked at other activities of this multifunctional viral protein. Buhlig et al. review the literature on the terminal protein region of HBV pol and argue that this highly-conserved, essential protein domain presents another potential drug target [13].

Therapeutic modulation of the antiviral immune response has also been proposed as a means to induce a cure for chronic HBV infection. One possible approach to boost antiviral immunity is to introduce constructs that express viral peptides in antigen-presenting cells to activate immune effector cells. Choga et al. explore the question [14] of which peptides should be used in this approach. In their study, they used in silico methods to correlate predicted Human Leukocyte Antigen Class II binding in African populations to viral peptides likely to be processed and presented, based on HBV strains endemic in Botswana (their target population). Here, they identified a number of candidate peptides that are well-conserved and are predicted to be displayed efficiently.

An alternative strategy for HBV cure is to de-silence anti-apoptotic signals in HBV-infected hepatocytes, causing cell death. Expression of cellular inhibitor of apoptosis proteins (cIAPs) can impair viral clearance by preventing TNF-mediated killing/death of infected cells. Smac mimetics (e.g., Birinapant) have been shown to antagonise cIAPs, increasing susceptibility to host CD4(+) T-cell responses and reducing HBV-infected cells [15]. However, these compounds can be secreted by efflux pumps in hepatocytes, reducing their efficacy. In this special issue, Morrish et al. show that by blocking hepatocyte efflux pumps with Zosuquidar in a HBV mouse model, Birinapant can more effectively facilitate the clearance of HBV-expressing cells [16]. Excitingly, this strategy could potentially increase the therapeutic window of Smac mimetics in humans and reduce any off-target drug effects.

*Implementation.* Research findings should ideally influence clinical practice, but there are many hurdles to their implementation. Liu and Chen recount the public health measures introduced since the 1970s in Taiwan, a country that had very high HBV prevalence [17]. These initiatives (including universal vaccination and reimbursement of anti-HBV treatments) effectively controlled and reduced virus transmission: Taiwan is now on track for HBV elimination.

In a complementary paper, Amerzhanov et al. review the past and current state of HBV prevalence across Central Asia [18]. As in Taiwan, increasing rates of childhood vaccination and facilitating access to antiviral therapies have reduced viral transmission, new infections and HBV-associated liver disease. Of course, most people with chronic HBV infection who were exposed at birth still have lifelong infections, so reducing HBV prevalence in a population takes many decades.

These reports serve as reminders that disease eradication is not only a scientific problem, but also a political one and broad-scale programs must be implemented to successfully eliminate the disease. We hope that political will and health structures advance further, so that when effective HBV cures are inevitably developed, they can be implemented widely and efficiently.

*Concluding remarks.* In conclusion, this Special Issue has touched on many of the pressing issues in HBV biology and clinical treatment. Nevertheless, these challenges are now being matched by rising enthusiasm in the field, and there is renewed hope that a cure is in sight. We thank all contributing authors and hope our readers enjoy this Special Issue as much as we have enjoyed editing it.
